# Postoperative Hypoxia and Length of Intensive Care Unit Stay after Cardiac Surgery: The Underweight Paradox?

**DOI:** 10.1371/journal.pone.0093992

**Published:** 2014-04-07

**Authors:** Marco Ranucci, Andrea Ballotta, Maria Teresa La Rovere, Serenella Castelvecchio, for the Surgical and Clinical Outcome Research (SCORE) Group

**Affiliations:** 1 Department of Cardiothoracic - Vascular Anesthesia and Intensive Care, Fondazione Salvatore Maugeri, IRCCS Istituto Scientifico di Montescano, Montescano, Italy; 2 Department of Cardiology, Fondazione Salvatore Maugeri, IRCCS Istituto Scientifico di Montescano, Montescano, Italy; Sapienza University of Rome, Italy

## Abstract

**Objective:**

Cardiac operations with cardiopulmonary bypass can be associated with postoperative lung dysfunction. The present study investigates the incidence of postoperative hypoxia after cardiac surgery, its relationship with the length of intensive care unit stay, and the role of body mass index in determining postoperative hypoxia and intensive care unit length of stay.

**Design:**

Single-center, retrospective study.

**Setting:**

University Hospital. Patients. Adult patients (N = 5,023) who underwent cardiac surgery with CPB.

**Interventions:**

None.

**Measurements and main results:**

According to the body mass index, patients were attributed to six classes, and obesity was defined as a body mass index >30. POH was defined as a PaO2/FiO2 ratio <200 at the arrival in the intensive care unit. Postoperative hypoxia was detected in 1,536 patients (30.6%). Obesity was an independent risk factor for postoperative hypoxia (odds ratio 2.4, 95% confidence interval 2.05–2.78, P = 0.001) and postoperative hypoxia was a determinant of intensive care unit length of stay. There is a significant inverse correlation between body mass index and PaO2/FiO2 ratio, with the risk of postoperative hypoxia increasing by 1.7 folds per each incremental body mass index class. The relationship between body mass index and intensive care unit length of stay is U-shaped, with longer intensive care unit stay in underweight patients and moderate-morbid obese patients.

**Conclusions:**

Obese patients are at higher risk for postoperative hypoxia, but this leads to a prolonged intensive care unit stay only for moderate-morbid obese patients. Obese patients are partially protected against the deleterious effects of hemodilution and transfusions. Underweight patients present the “paradox” of a better lung gas exchange but a longer intensive care unit stay. This is probably due to a higher severity of their cardiac disease.

## Introduction

Postoperative hypoxia (POH) is often detectable in patients after cardiac surgery with cardiopulmonary bypass (CPB) [1]–[4]. In the most severe cases, POH results in the prolongation of the mechanical ventilation time, intensive care unit (ICU) stay, need for tracheostomy, increased costs and resources utilization.

The classical pathophysiological pattern includes a decreased lung compliance, increased alveolo-arterial oxygen difference, increased intrapulmonary shunt fraction [2]–[4]. All these changes lead to a decreased ratio between the arterial oxygen tension (PaO_2_) and the inspiratory oxygen fraction (FiO_2_) (PaO_2_/FiO_2_).

Numerous factors – related to the baseline pulmonary function, the underlying disease and the operation itself- may be advocated to justify this finding, including increased alveolo-arterial membrane permeability, decreased pulmonary artery resistances, damages to the surfactant, fluid overload, and increased left atrial pressure [2]–[4].

The improvement of the techniques and materials of CPB [5]–[7] and the increased tendency towards normothermic or moderately hypothermic management during CPB [8] led to a decrease in the severity of lung complications after cardiac surgery. However there is still about 20%–25% of the patients [4], [9] who experience various degrees of lung dysfunction after cardiac surgery.

Among the risk factors for POH in cardiac operations, obesity plays a well-recognized role

[1], [10], [11]. However in many surgical operations, including cardiac surgery, obesity is not associated with a higher morbidity and mortality rate, and to some extent the outcome of obese patients may even be better, leading to what is known as the “obesity paradox” [12].

The aim of the present study is to investigate the relationship between body mass index and POH in adult cardiac surgery, with the primary endpoint of determining their respective association with postoperative ICU length of stay.

## Methods

This is a retrospective study based on the institutional database of the Cardiac Surgery Dept. of the IRCCS Policlinico San Donato. The study was conducted after approval of the Local Ethics Committee (Comitato Etico Ospedale San Raffaele, Milano, Italy; protocol approved on July 11^th^, 2013), and waiving of an informed consent. All the patients provided a written permission for the treatment of their data, in an anonymous form and for scientific purposes. This study was conducted in accordance with the amended Declaration of Helsinki.

### Patient population

The database is active since the year 2000. Starting in 2003, the database included (optional until 2011, and mandatory after that date) the value of PaO_2_ and FiO_2_ registered immediately after postoperative admission in the ICU, with the patient sedated, tracheally intubated, and mechanically ventilated. From these values, the PaO_2_/FiO_2_ ratio was assessed. At the initial investigation (February 2013), the database included 12,275 adult (18 years or more) patients. For 7,250 patients the PaO_2_/FiO_2_ ratio was not available, due to missing values of PaO_2_, FiO_2_, or both. The remaining 5,023 patients constituted the final patient population.

### Data collection and definitions

For each patient, the following data were collected and available:

Preoperative: demographics, with body mass index (BMI, weight in kilograms/height in meters^2^) calculation; BMI classification (6 stages) according to the World Health Organization [13]; left ventricular ejection fraction (%); recent (within 30 days prior to surgery) myocardial infarction; congestive heart failure; active endocarditis; peripheral vascular disease; unstable angina; serum creatinine value (mg/dL); serum bilirubin value (mg/dL); chronic obstructive pulmonary disease (chronic use of bronchodilators); diabetes (on medication); previous cerebrovascular accident; previous cardiac surgery; urgent procedure. Operative: type of operation; cardiopulmonary bypass (CPB) duration (minutes); lowest temperature (°C) on CPB; CPB circuit priming volume (mL/kg); nadir hematocrit on CPB (%). Postoperative: PaO_2_/FiO_2_ ratio and outcome measurements. Primary outcome endpoint was considered the ICU length of stay (days). Secondary outcome measurements included length of mechanical ventilation (hours), hospital stay (days); allogeneic blood products transfusions; surgical revision; low cardiac output (need for inotropic drugs >48 hours); myocardial infarction (new Q-waves + enzymatic criteria); atrial fibrillation (new onset); ventricular arrhythmias; stroke; acute kidney injury (peak postoperative serum creatinine double the baseline value); mesenteric infarction; mediastinitis; bloodstream infections (with positive cultures); need for tracheostomy; mortality (in-hospital or within 30 days from surgery after discharge).

POH was defined in case of a PaO_2_/FiO_2_ ratio <200 at the admission in the ICU.

Obesity was defined according to the World Health Organization as a BMI >30; BMI categories were defined according to the same source, as class 1 (underweight, BMI<18.5), class 2 (normal, BMI 18.5–24.9), class 3 (pre-obese, BMI 25–29.9), class 4 (mild obese, BMI 30–34.9), class 5 (moderate obese, BMI 35–35.9) and class 6 (morbid obese, BMI≥40) [13].

### Anesthesia, surgery and CPB management

Anesthesia was induced with an intravenous infusion of remifentanil and a midazolam or propofol bolus. Cisatracurium besylate or atracurium besylate was subsequently administered to allow tracheal intubation. Subsequently, the anesthesia was maintained with a continuous infusion of remifentanil and midazolam or propofol. Additionally, inhalatory agents (sevoflurane) were used based to the anesthesiologist's judgement.

The patients were mechanically ventilated with positive pressure, volume control, at a respiratory rate of 16 cycles per minute and a tidal volume of 6–7 mL/kg. The inhalatory gas mixture was initially settled at a 50∶50 oxygen:air ratio. Subsequent adjustments of the tidal volume and the FiO_2_ were applied to maintain the arterial carbon dioxide tension (PaCO_2_) between 30 mmHg and 35 mmHg, and the PaO_2_ between 100 and 200 mmHg.

Additional measures like positive end expiratory pressure (PEEP) were applied in case of unsatisfactory oxygenation.

Roller or centrifugal pumps for CPB were used according to the availability; a biocompatible treatment (phosphorylcholine coating) and a closed circuit with separation of the blood suctions was used in 20% of the patients. Since 2006, all the circuits were treated with a biocompatible coating.

During CPB, the lung was non ventilated, but a continuous low gas flow (FiO_2_ 0.50) was maintained. Before weaning from CPB, tracheal suction of bronchial secretions and recruiting manoeuvres through deep manual inflations were applied.

### Intensive care unit respiratory management

Initially, the patients were mechanically ventilated with the same parameters used intraoperatively. Subsequent adjustments of the mechanical ventilation setting were applied in order to maintain a correct lung gas exchange, acting on the respiratory rate, the tidal volume, the FiO_2_, and using adequate values of PEEP. Additional manoeuvers to optimize the patients oxygenation in case of poor PaO2/FiO2 ratio included recruitment manoeuvres, individualized setting of PEEP values, and decubitus changes (lateral and prone positions).

### Statistics

Comparisons between groups (POH vs. no POH) were performed using a Student's t test for continuous variables and a Pearson's chi-square test for categorical variables. Multivariable models were based on stepwise forward linear (continuous outcome variables) or logistic (binary outcome variables) regression analyses, producing odds ratios and 95% confidence interval when appropriate.

The association between the values of PaO_2_/FiO_2_ and the ICU length of stay, and between BMI and ICU length of stay were explored using polynomial (best fit) regression models with 95% confidence interval. All the data are expressed as mean ± standard error of the mean, or absolute numbers and percentage when appropriate. A p value <0.05 was consider significant for all the statistical tests. The statistical analysis was performed using the computer software SPSS 11.0 (SPSS; Chicago,IL) and GraphPad Prism 5.0 (GraphPad; La Jolla, CA).

## Results

One-thousand-five-hundreds-thirty six (1,536) patients fulfilled the definition of POH, representing 30.6% of the patient population.

The general characteristics of the patient population are depicted in table 1.

**Table 1:** Baseline variables of the patient population (N = 5,023).

Continuous variables	Mean (standard deviation)
Age (years)	66.3 (12.5)
Weight (kgs)	74.3 (13.8)
Body mass index	26.3 (4.2)
Hematocrit (%)	38.9 (4.8)
Left ventricular ejection fraction (%)	52.9 (12.1)
Serum creatinine (mg/dL)	1.08 (0.61)
Serum bilirubin (mg/dL)	0.70 (0.55)
Cardiopulmonary bypass duration (min)	82.2 (39.4)
Lowest temperature on cardiopulmonary bypass (°C)	31.7 (1.9)
Logistic EuroSCORE	6.6 (8.2)
**Categoric variables**	**Number (%)**
Gender male	3,456 (68.8)
Obesity	866 (17.2)
Unstable angina	208 (4.1)
Recent myocardial infarction	627 (12.5)
Congestive heart failure	325 (6.5)
Peripheral vascular disease	814 (16.2)
Active endocarditis	82 (1.6)
Chronic obstructive pulmonary disease	365 (7.3)
Previous cerebrovascular accident	153 (3.0)
Diabetes on medication	854 (17.0)
Non-elective surgery	169 (3.4)
Redo surgery	332 (6.6)
Isolated coronary surgery	2,075 (41.3)
Isolated mitral valve procedure	442 (8.8)
Any mitral valve procedure	1,133 (22.6)
Isolated aortic valve procedure	780 (15.5)
Combined coronary+valve	751 (15.0)
Others	604 (12.0)

Eight-hundred-sixty-six (17.2%) fulfilled the definition for obesity. Table 2 reports the distribution of the patient population according to the BMI classification, with the predicted (EuroSCORE) and the observed mortality rates. The preoperative mortality risk differed significantly (P = 0.015) only between normal patients and patients with mild obesity, whereas the observed mortality was significantly higher in underweight patients vs. normal, pre-obese, and mild obese patients.

**Table 2:** Patient distribution according to the body mass index class, with predicted (EuroSCORE) and observed mortality rates.

Body mass index class	Number (%)	Mortality rate (%)	
		Predicted % (95% confidence interval)	Observed % (95% confidence interval)
Underweight (<18.5)	103 (2.1)	7.0 (5.1–8.9)	6.8 (1.8 – 11.7)°
Normal (18.5–24.9)	1,968 (39.2)	7.1 (6.6–7.6)*	3.0 (2.2–3.7)
Pre-obese (25–29.9)	2,086 (41.5)	6.4 (5.9–6.8)	3.1 (2.4–3.9)
Mild obese (30–34.9)	711 (14.2)	5.7 (5.1–6.3)	2.6 (1.4–3.7)
Moderate obese (35–39.9)	128 (2.5)	5.6 (4.0–7.2)	4.7 (1.0–8.4)
Morbid obese (≥40)	27 (0.5)	6.2 (1.3–11.1)	0.0

* P = 0.015 vs. mild obese group; ° P<0.05 vs normal, pre-obese, mild obese groups.

Chronic obstructive pulmonary disease was identified in 365 (7.3%) patients; the incidence of chronic obstructive pulmonary disease was significantly (P = 0.001) higher in obese (10.6%) vs. non-obese (6.6%) patients. Hemodilution during CPB was significantly lower in obese patients, with a circuit priming volume of 8.4±2.3 mL/kg in obese patients and 10.7±3.1 mL/kg in non-obese patients (P = 0.001), leading to a nadir value of hematocrit on CPB significantly (P = 0.001) higher in obese patients (27.7±3.8) than in non-obese patients (26.4±3.7).

At the arrival in the ICU, the value of PaO_2_/FiO_2_ ratio was 282±123. One thousand five hundred thirty six (1,536) patients had a value below 200, representing 30.6% of the total patient population, and were identified as POH patients.

Table 3 reports the univariate and multivariable association between the pre and intraoperative variables of table 1 and the occurrence of POH. Independent risk factors for POH are age, serum creatinine level, obesity, diabetes, CPB duration, non-elective surgery, and coronary surgery of any kind. In the multivariable model BMI was excluded, due to its mathematical coupling and association with obesity.

**Table 3:** Univariate and multivariable association of pre and intraoperative variables with postoperative hypoxemia.

Continuous variables	Mean (standard deviation)			
	POH (N = 1,536)	No POH (N = 3,487)	P (univariate)	P (multivariable)
Age (years)	67.6 (11.2)	65.7 (12.9)	0.001	0.001
Serum creatinine (mg/dL)	1.13 (0.66)	1.06 (0.58)	0.001	0.022
Cardiopulmonary bypass duration (min)	86.0 (42.9)	80.6 (37.7)	0.001	0.001
Lowest temperature on cardiopulmonary bypass (°C)	31.6 (2.2)	31.7 (1.7)	0.037	0.414
Body mass index	27.7 (4.3)	25.7 (4.0)	0.001	----
**Categoric variables**	**Number (%)**			
Obesity	402 (26.2)	464 (13.3)	0.001	0.001
Diabetes on medication	316 (20.6)	538 (15.4)	0.001	0.009
Non-elective surgery	82 (5.3)	87 (2.5)	0.001	0.001
Isolated coronary surgery	688 (44.8)	1,387 (39.8)	0.001	0.117
Any coronary operation	1,005 (65.4)	2,033 (58.3)	0.001	0.001
Isolated mitral valve procedure	97 (6.3)	345 (9.9)	0.001	0.293
Any mitral valve procedure	314 (20.4)	819 (23.5)	0.017	0.815
Isolated aortic valve procedure	201 (13.1)	579 (16.6)	0.002	0.383
Combined coronary+valve	255 (16.6)	496 (14.2)	0.029	0.386

POH: postoperative hypoxemia.

Among these risk factors, obesity has the highest odds ratio for developing POH (2.4, 95% confidence interval 2.05–2.78).

The impact of POH on the ICU length of stay and other secondary outcomes is shown in table 4. After adjustment for the pre-operative confounders identified in table 1, POH remained an independent risk factor for a longer (about 1 day) ICU and hospital stay. Patients with POH demonstrated a higher rate of mediastinitis (odds ratio 4.6, 95% confidence interval 1.4–15.2), systemic blood infections (odds ratio 1.41, 95% confidence interval 1.02–1.94) and any infection (mediastinitis, systemic blood infections, surgical wound infections) (odds ratio 1.48, 95% confidence interval 1.08–2.02), with significantly higher peak values of serum creatinine and serum bilirubin.

**Table 4:** Crude and adjusted association of outcome variables with postoperative hypoxemia.

Continuous variables	Mean (standard deviation)			
	POH (N = 1,536)	No POH (N = 3,487)	P (crude)	P (adjusted^a^)
Intensive care unit stay (days)	4.1(6.5)	3.2 (4.4)	0.001	0.001
Postoperative hospital stay (days)	10.4 (8.41	9.5 (6.5)	0.001	0.004
Peak postoperative serum creatinine (mg/dL)	1.27 (0.89)	1.13 (0.75)	0.001	0.002
Peak postoperative serum bilirubin (mg/dL)	1.20 (1.9)	1.07 (0.92)	0.018	0.003
**Categoric variables**	**Number (%)**			
Prolonged mechanical ventilation	163 (10.6)	234 (6.7)	0.001	0.004
Low cardiac output	360 (23.4)	671 (19.2)	0.001	0.058
Acute kidney injury	72 (4.7)	106 (3.0)	0.004	0.095
Mediastinitis	8 (0.5)	4 (0.1)	0.007	0.013
Systemic blood infection	68 (4.4)	99 (2.8)	0.004	0.038
Any infection	76 (4.9)	103 (3.0)	0.001	0.007
Tracheostomy	27 (1.8)	31 (0.9)	0.008	0.082
Operative mortality	60 (3.9)	95 (2.7)	0.026	0.524

a adjustment for age, baseline serum creatinine, diabetes on medication, non-elective surgery, coronary surgery. POH: postoperative hypoxemia.

**Figure 1:**
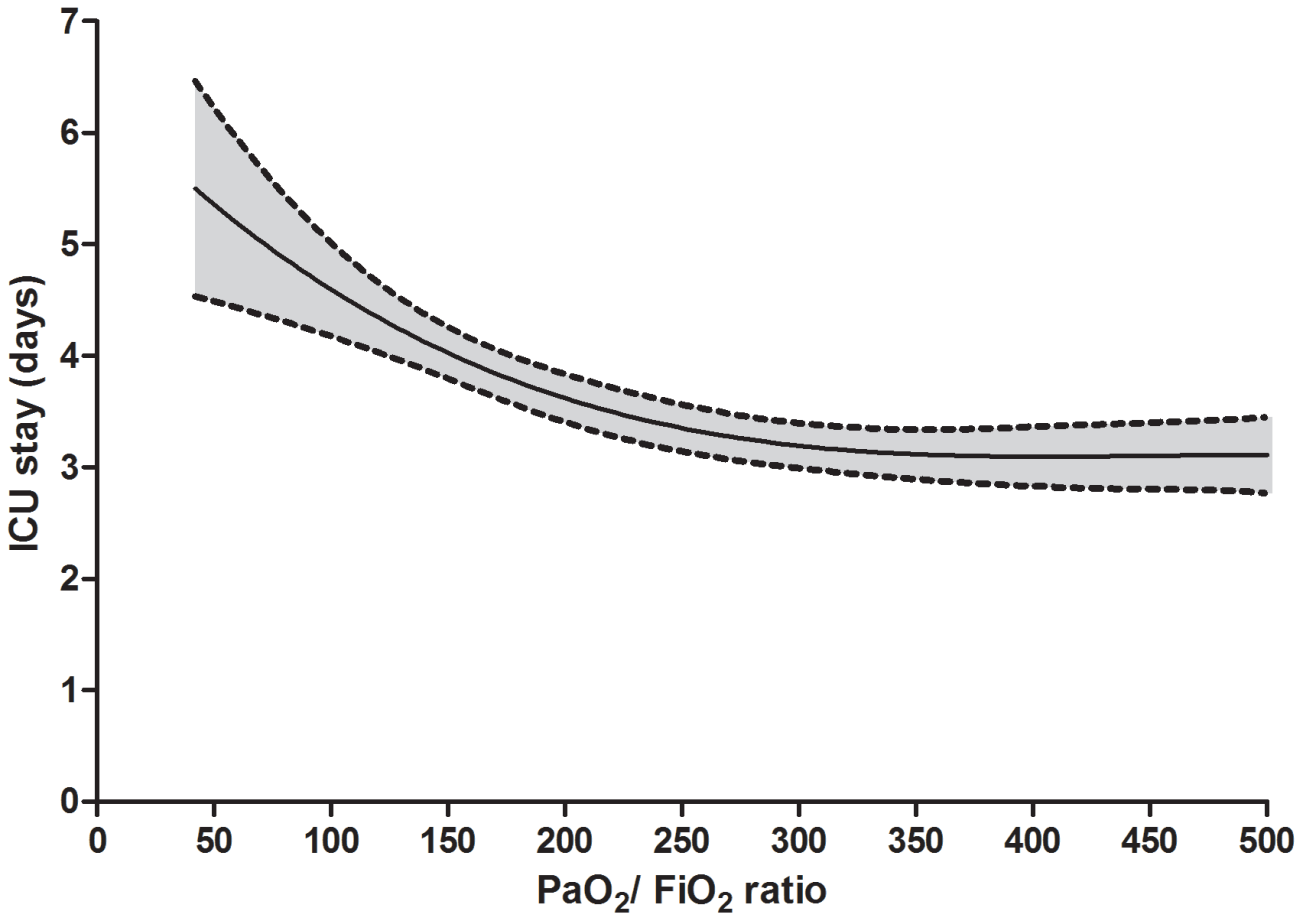
Univariate association between PaO_2_/FiO_2_ ratio at the arrival in the intensive care unit (ICU) and ICU length of stay. Grey area is 95% confidence interval.

Additional analyses were applied to investigate the association between POH and BMI on the ICU length of stay. In the overall population, we could identify a significant (P<0.001) cubic regression defining the association between PaO_2_/FiO_2_ ratio and ICU stay (figure 1).

**Figure 2:**
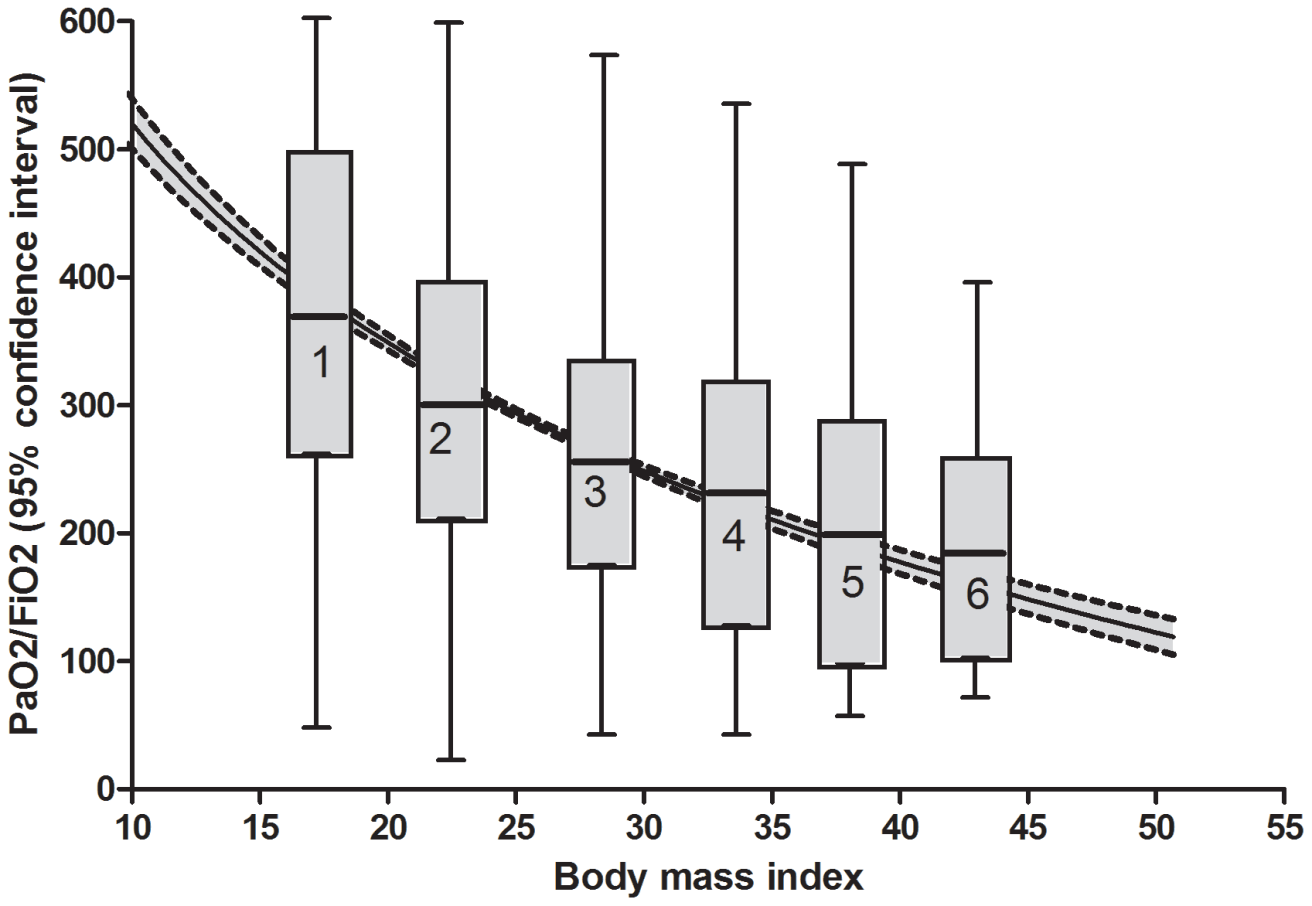
Univariate association between body mass index and PaO_2_/FiO_2_ ratio at the arrival in the intensive care. Grey area is 95% confidence interval. Boxes represent the six body mass index classes, with the median (line through the box), the interquartile range (box) and the 5% and 95% centiles (whiskers).

The BMI had a significant (P<0.001) logarithmic association with PaO_2_/FiO_2_ ratio (figure 2), with a progressive decrease of PaO_2_/FiO_2_ ratio for increasing level of BMI classification.

A logistic regression analysis for POH risk according to the BMI class found a significant (P<0.001) inverse association, with a relative risk for POH that is 1.73 (95% confidence interval 1.61–1.86) per each incremental BMI class.

**Figure 3:**
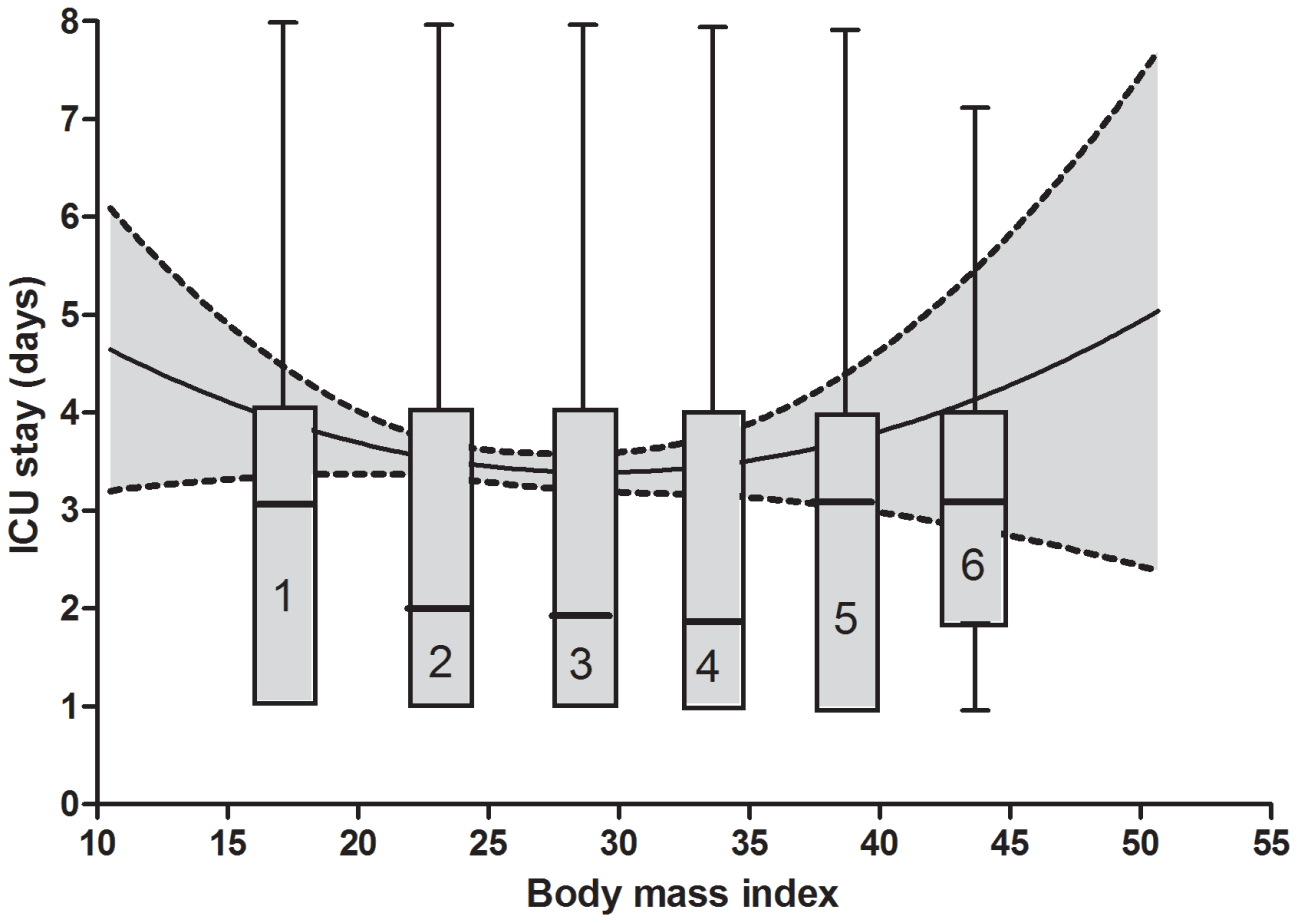
Univariate association between body mass index and intensive care unit (ICU) stay. Grey area is 95% confidence interval. Boxes represent the six body mass index classes, with the median (line through the box), the interquartile range (box) and the 5% and 95% centiles (whiskers).

The relationship of BMI with ICU length of stay is U-shaped (figure 3), with a significantly (P<0.01) longer ICU stay for underweight and moderate-morbid obese patients (median 3 days, interquartile range 3 days) vs. normal to mild obese patients (median 2 days, interquartile range 3 days).

Finally, given their recognized role in determining or deteriorating acute lung injury [12], transfusions of red blood cells, fresh frozen plasma, and platelets were compared between non-obese and obese patients (table 5). Obese patients had a significant lower transfusion rate for any transfusion, red blood cells, and fresh frozen plasma transfusions, and a trend towards a lower rate of platelet concentrates transfusions.

**Table 5:** Allogeneic blood products use in non-obese and obese patients.

Blood product	Non-obese (N = 4,157)	Obese (N = 866)	P
	Number (%)	Number (%)	
Any transfusion	1,938 (46.6)	322 (37.2)	0.001
Red blood cells	1,799 (43.3)	288 (33.3)	0.001
Fresh frozen plasma	494 (11.9)	82 (9.5)	0.042
Platelet concentrates	328 (7.9)	53 (6.1)	0.073

## Discussion

Our results confirm that POH is a common (30%) finding in cardiac surgery, and that obesity is an important determinant of POH, with a risk for POH in obese patients that is more than double the risk in non-obese patients. There is an inverse correlation between BMI and PaO_2_/FiO_2_ ratio, with a relative risk for POH that almost doubles for each increase in BMI class.

POH is an independent risk factor for a number of postoperative complications and longer ICU and hospital stay. Given the previous findings, it would be reasonable to hypothesize a direct relationship between BMI and ICU length of stay. Conversely, we could demonstrate that the longer ICU length of stay may be found at the extreme values of the BMI distribution, in correspondence with underweight (BMI<18.5) and moderate-morbid obese (BMI≥35) patients.

### Obesity, postoperative hypoxia, and outcome

Obesity and respiratory dysfunction are associated through a number of different mechanisms. First of all, the work of breathing is significantly higher in obese than in lean patients [14], [15], and the respiratory drive is blunted in morbid obese patients [16]. The main mechanism leading to the increased work of breathing is the decrease in functional residual capacity observed in obese patients [17]; additionally, an increase in total respiratory resistance is observed in supine position in obese vs. normal patients [18].

Other factors linking obesity and respiratory dysfunction include an impairment in respiratory muscle function, an increase in larynx collapsibility, local inflammation of the upper airways, and sleep breathing disorders due to neurohormonal influences [19]. Some of these factors, like respiratory drive, work of breathing, and upper airways conditions are practically absent in narcotized, paralyzed, and mechanically ventilated patients [20]. Others, including the changes in lung volumes, ventilation/perfusion mismatch, the increased total respiratory resistances and decreased thoraco-pulmonary compliance still play an important role even during anesthesia and mechanical ventilation.

After cardiac surgery with CPB, obese patients experience the combination of a pre-existing lung dysfunction (mainly represented by a volume reduction) with the perioperative damage that acts as an additional factor determining the loss of ventilating areas. The main pattern of this postoperative profile is extra-vascular lung water accumulation, triggered by the increased permeability of the alveolo-capillary membrane, hemodilution with fluid overload, and adverse effects of allogeneic blood product transfusions (including transfusion-associated lung injury).

In our obese patient population, the observed higher rate of patients with POH is likely to be explained by this pattern of chronic lung dysfunction plus acute insult. This condition leads to a significant prolongation of the ICU stay only for moderate-morbid obese patients. Pre-obese and mild obese patients present an increased incidence of POH, without a significant prolongation of the ICU stay. Among the factors that may temper the clinical effects of POH in obese patients is the significantly lower degree of intraoperative hemodilution and the significantly lower rate of transfusions.

The reason for these differences is the larger body mass index of obese patients, that leads to a lower hemodilution on CPB and higher values of hematocrit. From this point of view, obese patients are less prone to hemodilution-related extravascular lung water accumulation than non-obese patients. Allogeneic blood products transfusion is a well-recognized risk factor for postoperative lung dysfunction in cardiac surgery [21]–[24].

### Underweight, postoperative hypoxia, and outcome

Underweight patients have a prolonged ICU stay and even a significantly higher operative mortality despite better PaO_2_/FiO_2_ ratios and a lower rate of POH at the arrival in the ICU. This finding is more difficult to explain, and may represent an “underweight paradox”. As a matter of fact, there are many reasons to expect that underweight patients should suffer from POH after cardiac surgery. They are more prone to the deleterious effects of hemodilution and receive more allogeneic transfusions. Additionally, underweight is common in patients with chronic obstructive pulmonary disease [25], and is a risk factor for mortality in this group of patients for any degree of severity of the disease [26]. Despite this, in our series the underweight patients did demonstrate better values of PaO_2_/FiO_2_ ratio immediately after cardiac surgery.

The apparent paradox of a group of patients with better respiratory function but worse outcome is probably explained by a “reverse causation” mechanism [12], which means that underweight cardiac surgery patients have a number of additional co-morbidities, or a degree of severity of the cardiac disease, leading to a worse risk profile and worse outcome. The first possibility is however denied by our own data, where the underweight patients do not demonstrate a significantly higher risk profile with respect to normal or obese patients; it is therefore possible that underweight patients may have a higher severity of the underlying cardiac disease. In this setting, it is likely that the better lung gas exchange in underweight patients may offer little advantage in terms of clinical outcome.

However, we must recognize that underweight patients may actually have a higher non-cardiac risk profile related to conditions that are not included in the usual risk stratification (EuroSCORE II). It is likely that in the underweight class there are more frail patients, even if we can only hypothesize this, due to the lack of a frailty score in our database. Additionally, some underweight patients may suffer from a poor nutritional status. In a recent study, a preoperative screening of cardiac surgery patients with adequate nutritional status assessment tools demonstrated that a poor nutritional status is associated with worse outcomes [27].

The main strength of our study is the large patient population where we could investigate the PaO_2_/FiO_2_ ratio immediately after heart surgery. The main limitation is the absence of lung gas exchange data in the following days and after weaning from mechanical ventilation. An additional limitation is the absence of some potential determinants of lung dysfunction in our analysis (i.e. preoperative PaO2/FiO2 ratios, fluid balance, drug effects).

On a clinical basis, this study suggest that moderate-morbid obese patients should undertake (whenever feasible) specific programs before surgery, including respiratory rehabilitation, and diet and exercise to allow them to enter the mild obesity class, which is not associated with a prolonged ICU stay.
